# A comparative study for the organic byproducts from hydrothermal carbonizations of sugarcane bagasse and its bio-refined components cellulose and lignin

**DOI:** 10.1371/journal.pone.0197188

**Published:** 2018-06-01

**Authors:** Fang-Li Du, Qi-Shi Du, Jun Dai, Pei-Duo Tang, Yan-Ming Li, Si-Yu Long, Neng-Zhong Xie, Qing-Yan Wang, Ri-Bo Huang

**Affiliations:** 1 State key Laboratory of Bioenergy Enzyme Technology, National Engineering Research Center for Non-food Biorefinery, Guangxi Academy of Sciences, Nanning, Guangxi, China; 2 Gordon Life Science Institute, Belmont, MA, United States of America; Institute of Materials Science, GERMANY

## Abstract

Sugarcane bagasse was refined into cellulose, hemicellulose, and lignin using an ethanol-based organosolv technique. The hydrothermal carbonization (HTC) reactions were applied for bagasse and its two components cellulose and lignin. Based on GC-MS analysis, 32 (13+19) organic byproducts were derived from cellulose and lignin, more than the 22 byproducts from bagasse. Particularly, more valuable catechol products were obtained from lignin with 56.8% share in the total GC-MS integral area, much higher than the 2.263% share in the GC-MS integral areas of bagasse. The organic byproducts from lignin make up more than half of the total mass of lignin, indicating that lignin is a chemical treasure storage. In general, bio-refinery and HTC are two effective techniques for the valorization of bagasse and other biomass materials from agriculture and forest industry. HTC could convert the inferior biomass to superior biofuel with higher energy quantity of combustion, at the same time many valuable organic byproducts are produced. Bio-refinery could promote the HTC reaction of biomass more effective. With the help of bio-refinery and HTC, bagasse and other biomass materials are not only the sustainable energy resource, but also the renewable and environment friendly chemical materials, the best alternatives for petroleum, coal and natural gas.

## Introduction

Broadly speaking, biomass is all biologically-produced matters, including all plant and microorganism in lands and oceans. Each year large amount of biomass is produced from photosynthesis of plant, containing much more carbon than that consumed from fossil fuel [1]. More specially, biomass matters are the agricultural residues, including straw of wheat, rice, corn and other crops; and forest byproducts, such as saw dust, tree branch, bark, roots, and wood chips. Biomass is not only the largest renewable energy resource [2–4], but also the substainable resource for chemical industry [5,6] and material industry [7,8]. To deal with the global environmental and ecological problems, caused by over consuming of fossil fuel, including coal, petroleum, and natural gas, which lead to the global warming, frequently happened extreme weather and ecological disaster [9,10], biomass, particularly the waste from agriculture and forest industry, is the only way to replace coal and petroleum as both the energy fuel and as raw materials for chemical and material industries.

Biomass is complex organic matters that consist of a number of main organic macromolecules and special minor constituents, depending on different plant species. In order to utilize the biomass materials effectively, economically, and environment friendly, bio-refinery is an important, but difficult technique [11–13]. Usually the main organic macromolecules in biomass are cellulose, hemicellulose, and lignin. The goal of the bio-refinery is to separate these components to pure macromolecular compounds effectively without pollution.

**Fig 1:**
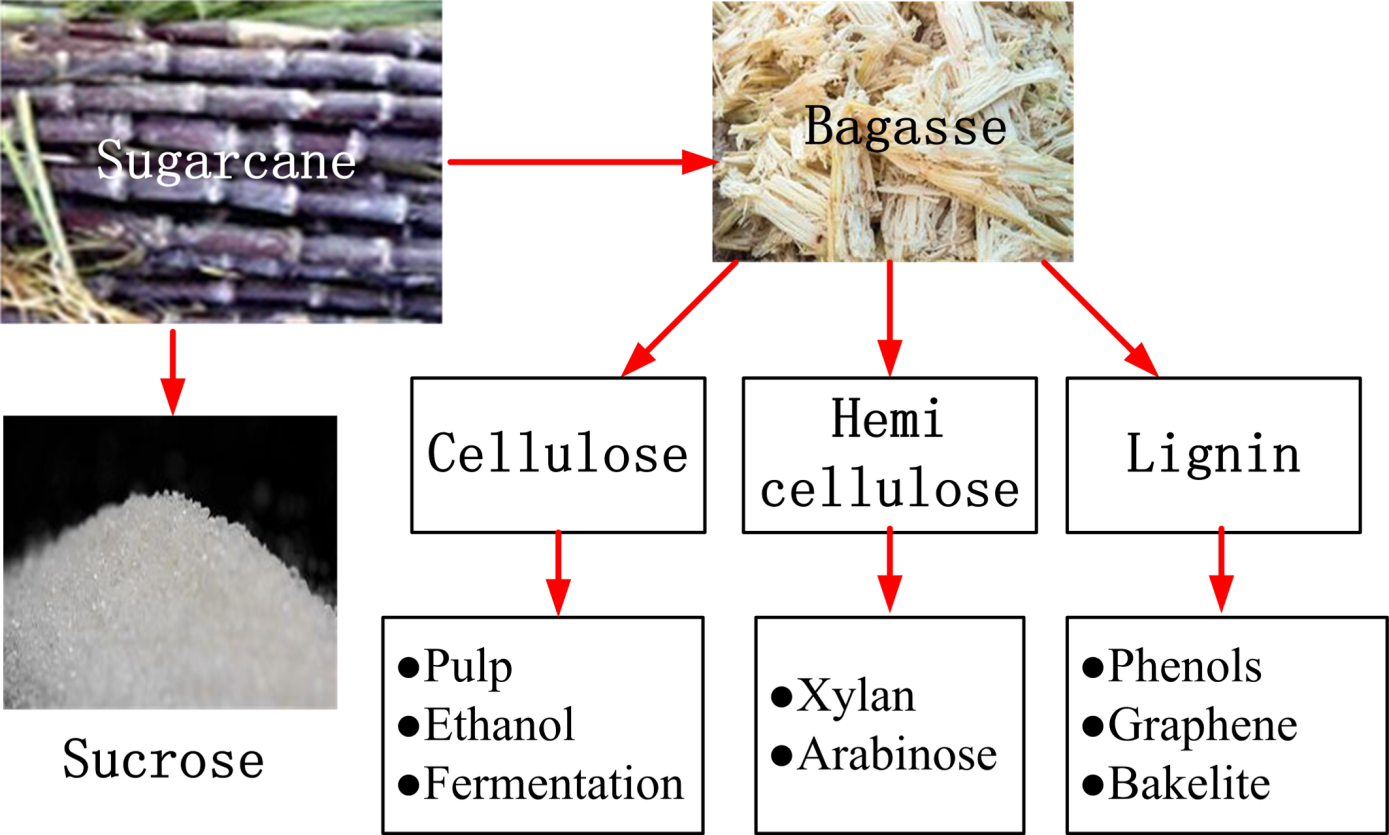
Illustration of bio-refinery of sugarcane bagasse. In above scheme the hemicellulose is used to prepare xylan, a very valuable medicine for hypertensive and diabetic patients. The cellulose is a widely-used material for pulp, glucose, and ethanol. The lignin is rich in phenolic compounds, the raw materials for many chemical products.

Sugarcane is produced in China, Brazil, Cuba, India, and many other countries in large scale. Bagasse is the residue of sugarcane after all sugar juice is squeezed and washed up by clean water for several times, which is the best raw material for development of bio-refinery technique. A bio-refinery scheme of sugarcane bagasse is illustrated in Fig 1.

In the above scheme the hemicellulose is used to prepare xylan, a very valuable medicine for hypertensive and diabetic patients [14], also a nutrition and healthy food product. The cellulose is a widely-used material for pulp, glucose, ethanol [12,15,16], and other products [17,18]. When ethanol is added in gasoline in 10 to 20% ratio, it can reduce the nitrogen oxides and 2.5 pm particles, discharged from automobiles, remarkably, which is one of the main sources for the severe air pollution and dust fog in north China [19–21]. The lignin is rich in phenolic compounds [22,23], the raw materials for many chemical products, such as phenolic plastics, carbon fiber, and binding agent for wood industry [24,25]. In this study a comparative study is conducted for the organic byproducts derived from the hydrothermal carbonizations (HTC) [26,27] of bagasse and its two refined components, cellulose and lignin, to explore what different organic products could be produced before and after bio-refinery of bagasse. The conclusions from this study would be helpful for the effective utilization of bagasse as the raw materials of chemical and material industry.

## Method and materials

The sugarcane bagasse used in this study was provided by a cane mill of Nanning sugar industry CO., LTD (http://www.nnsugar.com/) in Guangxi, China. The main components of sugarcane bagasse were comprehensively analyzed, and the general results were as follows: cellulose 45–55%, hemicellulose 20–25%, lignin 18–24%, ash 1–4%, and waxes <1, which is consistent with the reference [28].

### Bio-refinery of bagasse

**Fig 2:**
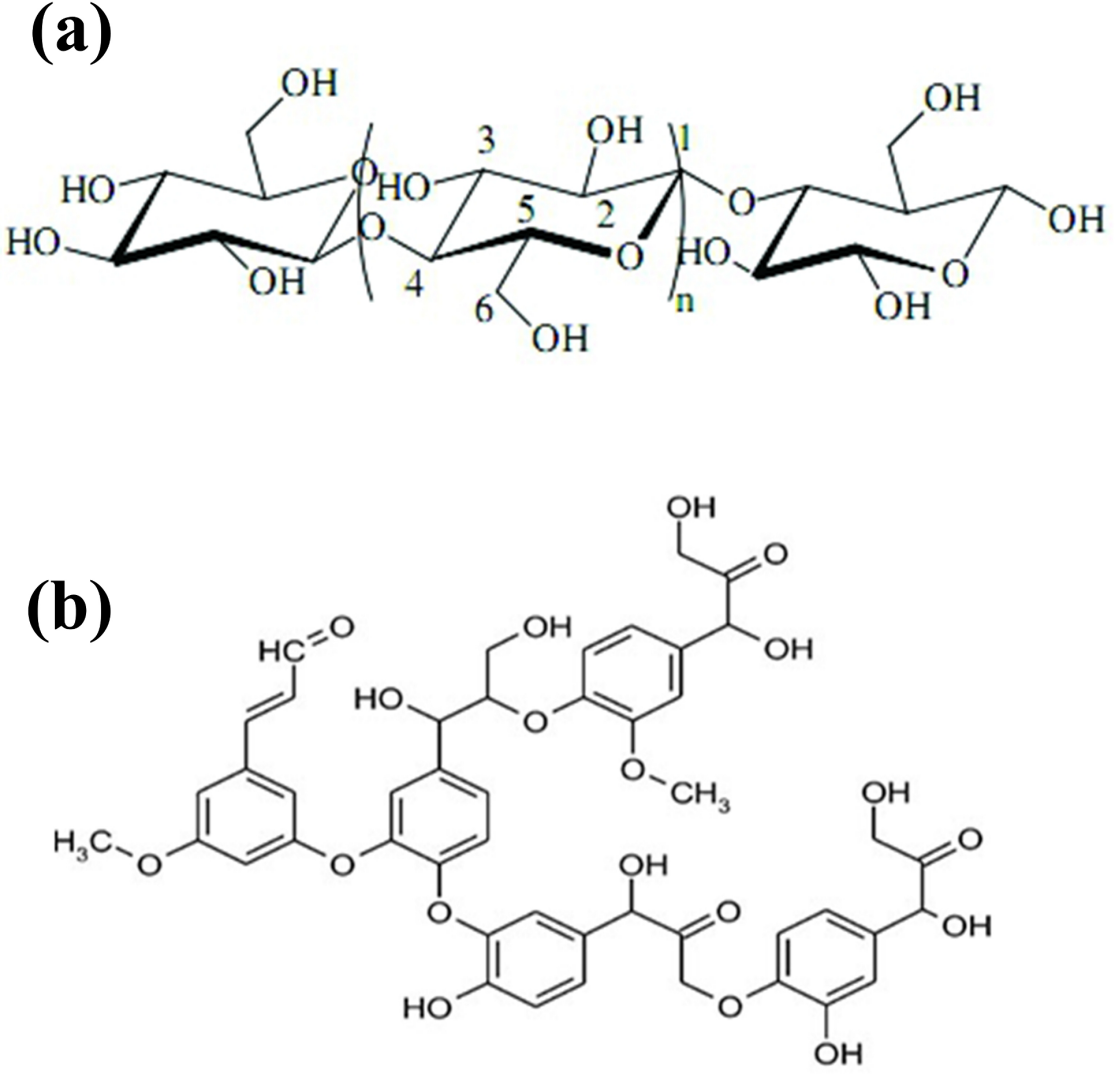
The chemical structures of cellulose and lignin. (a) Cellulose is a carbohydrate polymer consisting of glucose monomer through 1–4 glycoside bonds. (b) Lignin is cross linked phenolic polymer, consisting of three phenol monomers, coumaryl alcohol, coniferyl alcohol, and syringyl alcohol.

In this study the ethanol organosolv technique was used for the refinery of bagasse. Firstly clean and dried bagasse was grinded to 40 mesh (0.45mm) powder, then the bagasse powder was soaked into 55 wt% ethanol-water solution in pH = 3.0~4.0 condition, followed by heating to 180°C for 90 minutes in a special designed boiler. The dissolved lignin in ethanol-water solution was recovered by solvent vaporization. The undissolved cellulose and hemicellulose were removed from solution to a reactor, where in acidic condition the hemicellulos was hydrolyzed to xylose, and separated with cellulose. The chemical structures of cellulose and lignin are shown in Fig 2. Cellulose is a carbohydrate polymer consisting of glucose monomer through 1–4 glycoside bonds, while lignin is cross linked phenolic polymer, consisting of three phenol monomers, coumaryl alcohol, coniferyl alcohol, and syringyl alcohol.

### Hydrothermal carbonization reactions

Usually hydrothermal carbonizations (HTC) of biomass are used to convert the biomass materials to higher calorific biofuel [29–32]. In HTC reaction elevated temperatures (180–220°C) is applied to biomass in a suspension with water under saturated pressure for several hours. With this conversion process, a lignite-like, easy to handle biofuel with higher calorific and well-defined properties can be created [6,33]. At the same time many useful organic compounds are produced as byproducts [26,34,35]. In this study we focus on the different organic products obtained from the HTC reactions of the three biomass materials, bagasse and its two refined components, cellulose and lignin.

**Fig 3:**
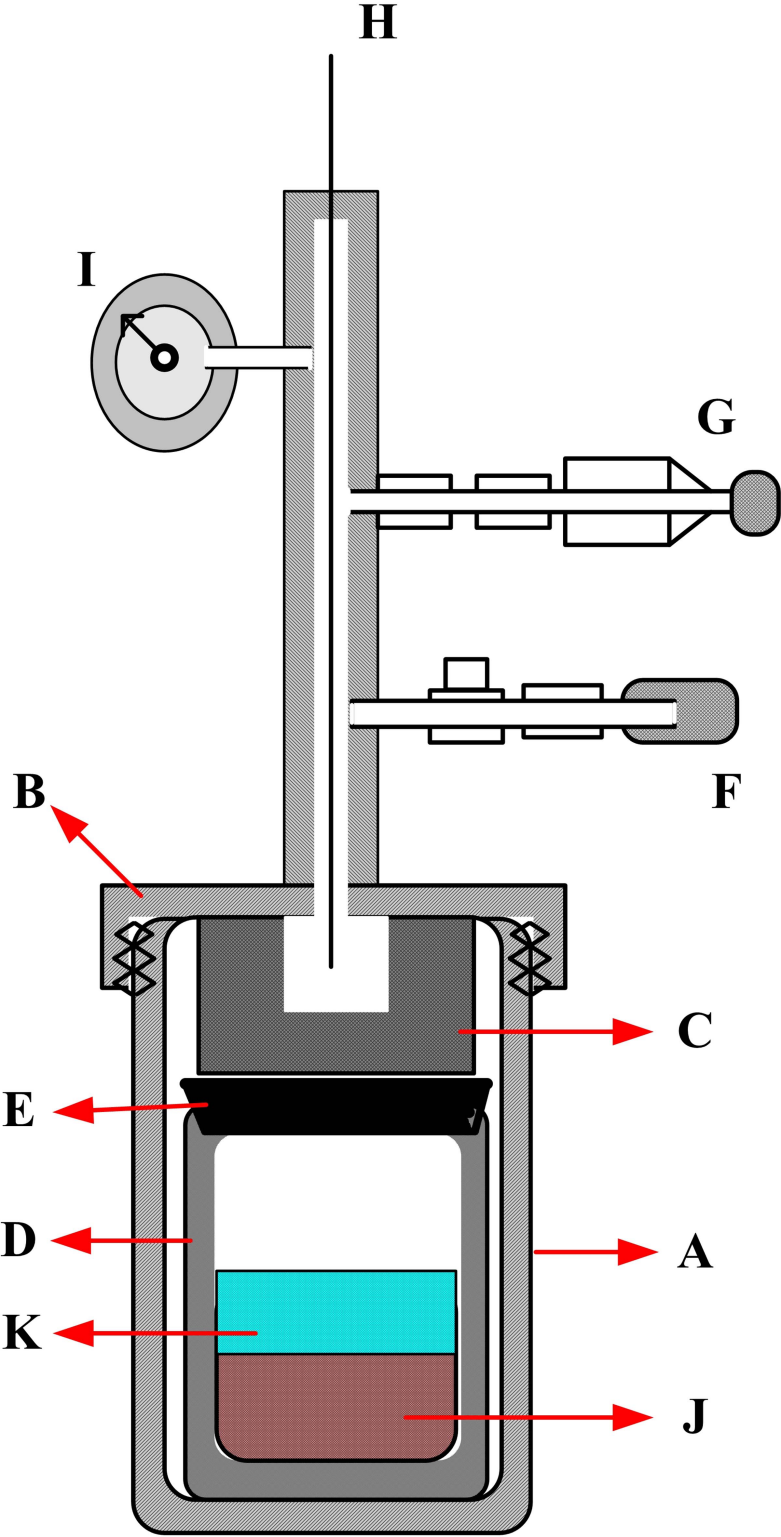
The structure of hydrothermal carbonization (HTC) reactor. A: Stainless steel vessel, B: pressure vessel closure, C: Fixing iron, D: Teflon cylinder, E: Teflon seal cover, F: Pressure valve, G: Relieve valve, H: Thermocouple, I: Pressure gauge, J: Biomass material, K: Water.

The HTC experimental runs of the three biomass materials were performed in a batch Teflon-lined autoclave boiler with 100 ml volume. The HTC reactor is a stainless steel cylinder 11.5 cm high, 5.5 cm inside diameter, and 150 ml capacity. A 2.5-kW muffle furnace is used for heating the reactor. The reactor is fitted with temperature and pressure sensors, as well as pressure relieve valve, as shown in Fig 3.

### Gas chromatography/mass spectrometry analysis

After hydrothermal reaction, the liquid organic products were extracted by ethyl acetate, then analyzed by gas chromatography/mass spectrometry (GC-MS) using Agilent 7890A /5973N series, equipped with a HP-5MS (30 m×0.25 mm×0.25 um) column [36,37]. Before analysis, the samples were filtered by a 0.45 μm filter to remove particles. Each time a 2 μL sample was injected into the GC-MS system at initial column temperature 45℃, holding for 1 min, ramped to 150°C with 4°C/min; holding for 3 min, ramped up to 280°C with 9°C/ min; holding for another 5 min. The injector was kept at 250°C in spit mode (10:1) with the carrier gas helium. The ion source was maintained at 230°C. Mass spectrometric measurements were performed using electron impact ionization (EI) at an ionizing voltage 70 eV, and a scanning range of m/z 50–550. Peak identification was accomplished by comparing mass spectra to the mass spectral library of National Institute of Standards and Technology (NIST) 2011 (https://www.nist.gov/) [36–39].

## Results

The HTC reactions of three biomass materials are performed at three temperatures (200, 250, and 300°C). Each time 10 grams dried material and 50 grams deionized water were added in a 100 ml Teflon autoclave reactor. After HTC reactions the organic products in the water solutions were analyzed using GC-MS instrument. The physical conditions of HTC reactions and the yields of solid carbon and organic byproducts are listed in Table 1. The yield of organic products is estimated by subtracting the carbon yield from the total mass of biomass material.

**Table 1:** Physical conditions of HTC reactions and yields of organic products and solid carbon.

**Bagasse**					
Temperature (°C)	Pressure (mPa)	Water (g)	Bagasse (g)	Organic products ^a^	Carbon yield ^b^
200	108	50	10	51%	46%
250	121	50	10	55%	42%
300	131	50	10	49%	48%
**Cellulose**					
Temperature (°C)	Pressure (mPa)	Water (g)	Cellulose (g)	Organic products ^a^	Carbon yield ^b^
200	108	50	10	57%	40%
250	121	50	10	52%	45%
300	131	50	10	43%	54%
**Lignin**					
Temperature (°C)	Pressure (mPa)	Water (g)	Lignin (g)	Organic products ^a^	Carbon yield ^b^
200	108	50	10	66%	31%
250	121	50	10	64%	33%
300	131	50	10	56%	41%

^a^ The yield of organic products are estimated by subtracting the carbon yield from the total mass of biomass.

^b^ There is a ~3% mass lose because of the escape of volatile organic products from water solution.

### Organic products from HTC reactions of bagasse

**Fig 4:**
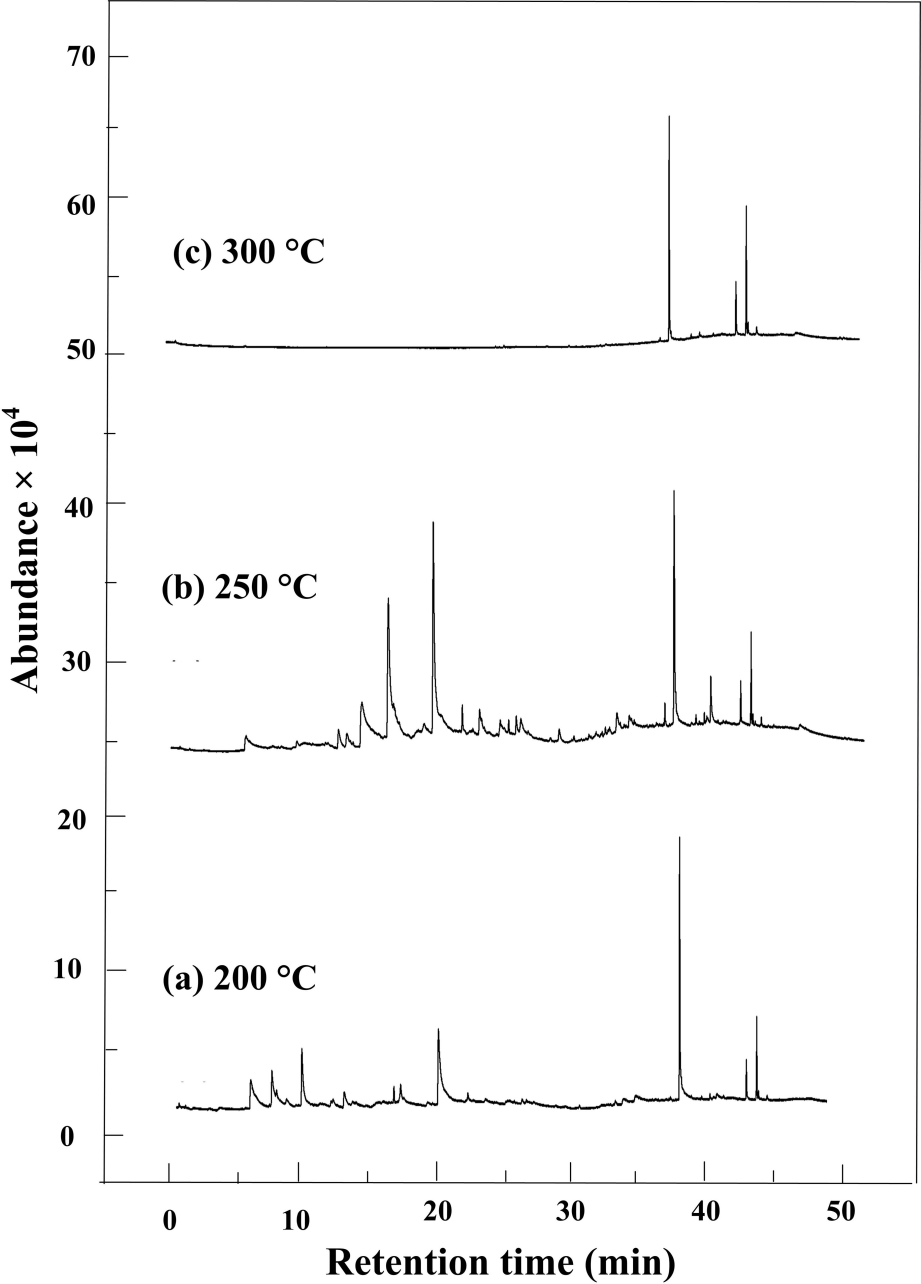
GC-MS spectra of organic byproducts from HTC reactions of bagasse at three temperatures. (a) 200, (b) 250,(c) 300°C.

The GC-MS spectra of organic byproducts from HTC reactions of bagasse at three temperatures (200, 250, and 300°C) are shown in Fig 4.

Based on the GC-MS analysis, a total of 11, 22 and 4 organic compounds were identified when the HTC reactions were maintained at 200, 250 and 300°C, respectively. With the temperature increasing from 200°C to 300°C, the number of compounds increases firstly, and then decreased. When the temperature was elevated to 300°C, the compounds mainly were observed after 30 min residence time. The detailed compounds are listed in Table 2. The compounds were classified as phenols, aromatic hydrocarbons, and some other oxygen-containing compounds, such as alcohols, ketones, acids and esters.

**Table 2:** Liquid organic products from HTC reactions of bagasse.

Retention time/min	Compounds	Share of products in liquefaction (area %)		
		200°C	250°C	300°C
8.492	Phenol	1.848	1.355	-
9.803	2-hydroxy-3-methyl-2-cyclopenten-1-one	9.764	-	-
10.108	pyrazole-5-carboxylic acid	1.874	-	-
11.748	phenol, 2-methoxy-	14.742	-	-
14.518	phenol, 4-ethyl-	4.053	3.096	-
15.029	2-acetonylcyclopentanone	-	0.327	-
15.99	catechol	-	2.263	-
17.742	naphthalene, 2,6-bis(1,1-dimethylethyl)-	1.757	-	-
17.746	1,2-benzenediol, 3-methoxy-	-	24.437	-
18.175	phenol, 4-ethyl-2-methoxy-	3.349		-
20.635	phenol, 2,6-dimethoxy-	25.309	28.660	-
22.556	3,5-dimethyl-1-dimethylphenylsilyloxybenzene	-	1.885	-
23.681	1,2,3-trimethoxybenzene	-	3.673	-
25.025	phenol, 2,3,5-trimethyl-	-	0.951	-
25.578	phenol, 2,5-bis(1,1-dimethylethyl)-	-	0.775	-
26.064	4-ethylbiphenyl	-	1.350	-
26.373	benzeneacetic acid, .alpha.-hydroxy-2-methoxy-	-	0.286	-
28.859	1-(2,5-dimethoxyphenyl)-propanol	-	0.898	
32.622	ethanone, 1-(4-hydroxy-3,5-dimethoxyphenyl)-	-	1.377	-
33.43	desaspidinol	-	0.218	-
35.738	7,9-di-tert-butyl-1-oxaspiro(4,5)deca-6,9-diene-2,8-dione	-	1.150	-
36.328	dibutyl phthalate	27.509	16.583	53.603
36.443	n-Hexadecanoic acid	-	1.505	-
38.306	methyl stearate	-	0.465	-
38.727	octadecanoic acid	-	3.393	-
40.672	adipic acid dioctyl ester	3.335	1.711	13.544
41.348	phenol, 2,2'-methylenebis[6-(1,1-dimethylethyl)-4-methyl-	6.460	3.635	29.693
41.463	phenol, 2,4-bis(1-phenylethyl)-	-	-	3.159

From Table 2 we can see that the hydrothermal products of bagasse at 200°C were mainly the phenolic compounds, making up 55.761% of the total integral areas. Among the phenolic compounds, the major chemicals were 2, 6-dimethoxy-phenol, 2-methoxy-phenol and 2, 2'-methylenebis[6-(1,1-dimethylethyl)-4-methyl-phenol, up to 46.511% of the total integral areas. The second compound group was esters, including dibutyl-phthalate and adipic acid dioctyl ester, possessing 30.844% of the total integral areas. The other compound groups were ketones, aromatic hydrocarbons, and organic acids with smaller integral areas.

When the reaction temperature was 250°C, more compounds were obtained from HTC reactions of bagasse. The portion of phenolic compounds increased to 65.39% of the total integral area. The major phenolic compounds were 2, 6-dimethoxy-phenol, 3-methoxy-1, 2-benzenediol, 4-ethyl- phenol, and 2,2'-methylenebis[6-(1,1-dimethylethyl)-4-methyl-phenol, whose integral areas were 28.660%, 24.437%, 3.096%, 3.635%, respectively. In addition to phenolic compounds, a number of esters, ketones, aromatic hydrocarbons, acids and alcohols were also detected. Distinctly, some new compound structures were observed when the HTC temperature was 250°C, such as catechol, 3-methoxy-1, 2-benzenediol, 4-ethylbiphenyl, and octadecanoic acid, etc.

As the HTC reaction temperature increased to 300°C, the GC-MS analysis result of the bagasse HTC products displayed three distinct features. (1) Among the phenolic compounds only 2,2'-methylenebis[6-(1,1-dimethylethyl)-4-methyl- phenol and 2,4-bis(1-phenylethyl)- phenol were observed. Other phenolic compounds, such as 4-ethyl- Phenol and 2,6-dimethoxy- phenol, disappeared. (2) Very high integral areas of dibutyl phthalate and adipic acid dioctyl ester were detected, accounted for the 53.603% and 13.544% of the total integral areas, respectively. (3) The ketones, acids and aromatic hydrocarbons were not found at 300°C.

### Organic products from HTC reactions of cellulose

**Fig 5:**
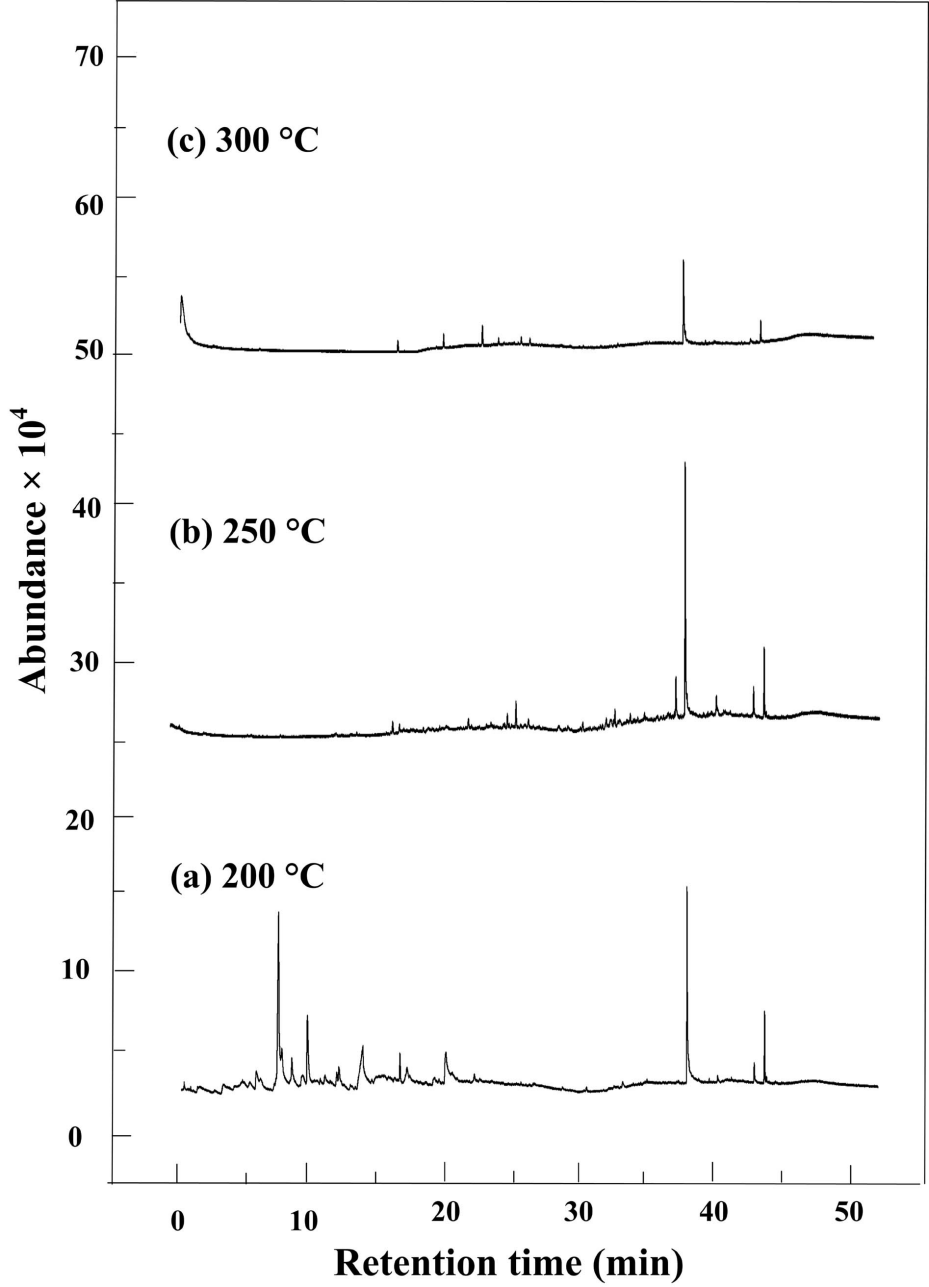
GC-MS spectra of organic byproducts from HTC reactions of cellulose at three temperatures. (a) 200, (b) 250,(c) 300°C.

The GC-MS spectra of cellulose’s HTC products at three temperatures (200, 250, and 300°C) are shown in Fig 5.

By GC-MS analysis, a total of 13, 5 and 3 compounds were identified when the temperature was maintained at 200, 250 and 300°C, respectively. With the increase of temperature, the number of detected compounds decreased. When the temperature was 250 and 300°C, the compounds mainly were found after 25 min residence time. The detailed information of compounds is listed in Table 3. The compounds were classified as phenols, aromatic hydrocarbons, alkanes, and some oxygen-containing compounds, such as alcohols, ketones, acids and esters.

**Table 3:** Liquid organic products from HTC reactions of cellulose.

Retention time/min	Compounds	Share of products in liquefaction (area %)		
		200°C	250°C	300°C
8.434	phenol	1.597	-	-
9.881	2-cyclopenten-1-one, 2-hydroxy-3-methyl-	31.855	-	-
10.083	2-methyl-2,3-divinyloxirane	7.127	-	-
10.73	2-cyclopenten-1-one, 3-ethyl-2-hydroxy-	4.587	-	-
11.74	phenol, 2-methoxy-	10.950	-	-
13.776	exo-norborneol, methyl ether	2.233	-	-
15.339	benzoic acid	10.940	-	-
17.738	naphthalene, 2,6-bis(1,1-dimethylethyl)-	2.512	-	-
20.709	phenol, 2,6-dimethoxy-	6.044	-	-
23.236	benzene, 1-methyl-3,5-bis[(trimethylsilyl)oxy]-	-	-	12.650
25.569	phenol, 2,4-bis(1,1-dimethylethyl)-	-	7.969	-
35.734	7,9-di-tert-butyl-1-oxaspiro(4,5)deca-6,9-diene-2,8-dione	-	10.379	-
36.324	dibutyl phthalate	15.614	57.947	40.509
36.439	n-Hexadecanoic acid	1.006	-	-
40.676	adipic acid dioctyl ester	1.608	9.259	-
41.348	phenol, 2,2'-methylenebis[6-(1,1-dimethylethyl)-4-methyl-	3.927	14.446	8.729

When the HTC temperature was 200°C, the products mainly distributed at 8–42 min residence time in the GC-MS spectra. In the HTC products of cellulose the dominant chemical components were ketone compounds, such as 2-hydroxy-3-methyl-2-cyclopenten-1-one and 3-ethyl-2-hydroxy-2-cyclopenten-1-one, which made up 36.442% of the total integral areas. In addition to ketone compounds, other oxygen-containing compounds were also found, such as esters, acids, and ethers. Various types of phenolic compounds, including 2-methoxy- phenol, 2,6-dimethoxy- phenol, 2,4-bis(1,1-dimethylethyl)- phenol and 2,2'-methylenebis[6-(1,1-dimethylethyl)-4-methyl- phenol were analyzed simultaneously, which shared 22.518% of the total integral areas.

As the temperature increased to 250°C, the ketone compound 2-hydroxy-3-methyl-2-cyclopenten-1-one, which abounded at 200°C, was not found, however, a new ketone compound 7,9-di-tert-butyl-1-oxaspiro(4,5)deca-6,9-diene-2,8-dione was detected at 250°C, which accounted for 10.379% of the total integral areas. Only two phenolic compounds, namely 2, 4-bis(1,1-dimethylethyl)- phenol and 2,2'-methylenebis[6-(1,1-dimethylethyl)-4-methyl- phenol, were founded, and the relative content of the latter increased from 3.927% to 14.446%. The relative content of ester compound dibutyl phthalate increased to 57.947% of the total integral areas, which was much higher than 15.614% at 200°C.

More remarkably, only three compounds were found as the HTC temperature increased to 300°C. Besides the similar compounds dibutyl phthalate and 2, 2'-methylenebis[6-(1,1-dimethylethyl)-4-methyl- phenol, a new aromatic hydrocarbon compound 1-methyl-3,5-bis[(trimethylsilyl)oxy]- benzene was detected, which accounted for 12.650% of the integral areas.

### Organic products from HTC reactions of lignin

**Fig 6:**
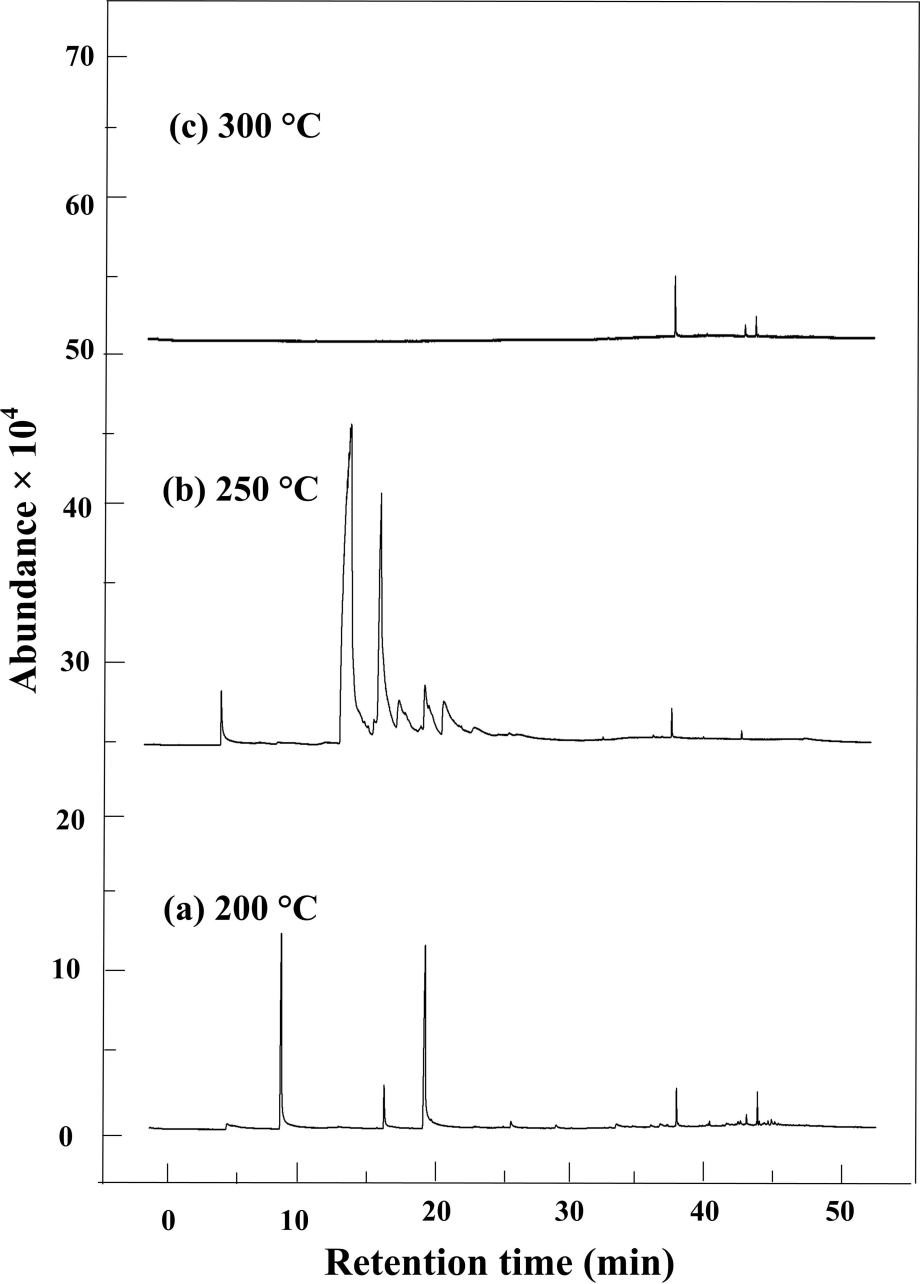
GC-MS spectra of organic byproducts from HTC reactions of lignin at three temperatures. (a) 200, (b) 250, (c) 300°C.

The GC-MS spectra of hydrothermal products of lignin at three HTC temperatures (200, 250, and 300°C) are summarized in Fig 6.

Based on GC-MS analysis, total of 19, 14 and 3 compounds were identified when the HTC temperature maintained at 200, 250 and 300°C, respectively. As shown in Fig 5, when the temperature increased to 300°C, the compounds only were observed after 30 min residence time. This phenomenon was similar with that of bagasse and cellulose. The detailed compounds are listed in Table 4.

**Table 4:** Liquid organic products from HTC reactions of lignin.

Retention time/min	Compounds	Share of products in liquefaction (area %)		
		200°C	250°C	300°C
8.381	phenol	0.614	1.835	-
11.814	phenol, 2-methoxy-	35.832	-	-
15.916	catechol	-	56.751	-
18.179	phenol, 4-ethyl-2-methoxy-	5.972	-	-
18.319	1,2-benzenediol, 3-methoxy-	-	26.196	-
19.44	1,2-benzenediol, 4-methyl-	-	5.675	-
20.742	phenol, 2,6-dimethoxy-	41.854	-	-
20.776	2-(phenylthio)ethanol	-	0.377	-
20.907	phenol, 2,6-dimethoxy-	2.911	5.259	-
21.447	phenol, 3,4-dimethoxy-	-	1.178	-
22.177	4-ethylcatechol	-	2.063	-
24.069	1-(2,4-dimethyl-furan-3-yl)-ethanone	-	0.051	-
24.102	benzene, 2-fluoro-1,3,5-trimethyl-	-	0.010	-
24.135	benzeneacetic acid, 3,4-dihydroxy-	-	0.003	-
26.052	5-tert-butylpyrogallol	1.427	-	-
28.854	1-methylpyridin(2H)-2-one-5-carboxylic acid, methyl ester	0.641	-	-
32.078	1-dimethylphenylsilyloxy-3-methylbenzene	-	0.053	-
32.601	ethanone, 1-(4-hydroxy-3,5-dimethoxyphenyl)-	0.502	-	-
35.318	bicyclo[4.4.0]dec-1-ene, 2-isopropyl-5-methyl-9-methylene-	0.406	-	-
36.328	dibutyl phthalate	3.340	0.429	65.242
36.443	terephthalic monohydroxamic acid	0.212	-	-
38.376	2-(4-Methoxyphenyl)-5-morpholin-4-yl-3H-imidazol-4- carboxamide	0.352	-	-
40.145	2,4-dimethyl-indeno[1,2-g]quinolin-10-one	0.487	-	-
40.297	7H-furo[3,2-g][1]benzopyran-7-one, 2-(1-hydroxy-1-methylethyl)-4-methoxy-	0.497	-	-
40.672	adipic acid dioctyl ester	0.718	0.120	13.962
41.348	phenol, 2,2'-methylenebis[6-(1,1-dimethylethyl)-4-methyl-	2.376	-	20.796
41.464	phenol, 2,4-bis(1-phenylethyl)-	0.627	-	-
42.214	2,2'-isopropylidenebis(3-methylbenzofuran)	0.828	-	-
42.428	2-(10-methyl-10H-acridin-9-ylideneamino)-phenol	0.403	-	-

When the hydrothermal temperature was 200°C, the mainly identified chemical compounds were phenols, esters, and ketones, and the low-content compounds were acids, furans, amides, and alkenes. The relative content of phenols was high up to 92.016%, including 2,6-dimethoxy- phenol, 2-methoxy- phenol, 4-ethyl-2-methoxy- phenol, 2,6-dimethoxy- phenol, and 2,2'-methylenebis[6-(1,1-dimethylethyl)-4-methyl- phenol. The total content of esters and ketones were 4.699% and 1.486% of the integral areas, respectively.

The once main compounds 2,6-dimethoxy- Phenol, 2-methoxy- phenol and 4-ethyl-2-methoxy- phenol at 200°C were not found at 250°C, however, two di-phenol compounds catechol, 3-methoxy-1,2-benzenediol and 4-methyl-1,2-benzenediol, which were not observed at 200°C, were achieved at 250°C, and accounted for the largest content. Acids, furans, amides and alkenes were not found at 250°C.

The composition of the chemicals became simple as the temperature increased to 300°C. The only main compound was dibutyl phthalate, whose shares in all organic products increased to 65.242%, much higher than that at 200 and 250°C. The contents of 2,2'-methylenebis[6-(1,1-dimethylethyl)-4-methyl- phenol and adipic acid dioctyl ester were also higher than that at 200 and 250°C.

## Discussion

Based on the experimental results it was observed that many valuable organic products could be produced from the HTC reactions of bio-refined components of sugarcane bagasse. Temperature of HTC reactions has significant impact on the chemical composition of the organic byproducts. Many types of organic compounds were produced at the temperature range of 200 to 250°C for the HTC reactions of bagasse with its component (cellulose and lignin). As the temperature increased to 300°C, the various types of organic products derived from the HTC reactions, decreased gradually. This is as a result of higher molecular polymeric compound which couldn’t be detected via GC-MS, thus increase in temperature consequently lead to less identified organic species. Also at 300°C, the hydrothermal organic products of bagasse, cellulose and lignin shared a common character: dibutyl phthalate was the dominant component, meaning that at higher temperature, esterification was the dominant reaction, which allows the active fragments of acids to react with phenols or alcohols and form ester compounds. The most abundant compounds, derived at 250°C from the HTC reaction of bagasse were phenolic-related componds, which was also observed at the reaction of lignin at 200°C, 50°C lower than the bagasse. It has been reported that the phenolic compounds mainly forms from the breakage of β-aryl and benzoylether bonds in lignin and further decomposes to phenolic products, such as benzenediols [40]. In bagasse, the lignin present was chemically connected with cellulose by chemical bonds and it requires higher energy to make these bonds to rupture, so the reaction temperature was higher than that of lignin. Unlike bagasse and lignin, the most abundant compounds, derived from cellulose, were ketones, followed by the esters, and finally the phenolic compounds were the lowest components.

In the HTC reactions of bagasse and its two refined components (cellulose and lignin), more organic byproducts (13+19 compounds) were produced from cellulose and lignin than that from bagasse (22 compounds). Particularly, high valuable catechol products, around US$ 2000~3000/ton [41], were obtained from the HTC reaction of lignin at 250°C with 56.8% production shares in the total GC-MS integral areas. In contrast, the GC-MS integral area of catechol products from the bagasse HTC reaction was only 2.263%. In HTC reaction the organic byproducts from lignin make up more than half of the total mass of lignin, indicating that lignin is a chemical treasure storage [42].

Finally in general, bio-refinery and HTC are two effective techniques for the valorization of bagasse and other biomass materials from agricultural that are environmental pollutants in many cases [32,43–45]. HTC could convert the inferior biomass to superior biofuel with higher energy quantity of combustion, at the same time many valuable organic byproducts will be produced. Bio-refinery makes the HTC reaction of biomass more effective. With the help of bio-refinery and HTC, bagasse and other biomass materials are not only the sustainable energy resource, but also the renewable and environment friendly chemical materials, the best alternatives for petroleum, coal and natural gas.
